# Transport of Neutral Amino Acids in the Jejunum of Pigs with Special Consideration of L-Methionine

**DOI:** 10.3390/nu16193418

**Published:** 2024-10-09

**Authors:** Isabel I. Schermuly, Stella Romanet, Amlan K. Patra, Lucia Mastrototaro, Andreas Lemme, Robert Pieper, Jürgen Zentek, Jörg R. Aschenbach

**Affiliations:** 1 Institute of Veterinary Physiology, Freie Universität Berlin, Königsweg 56, 14163 Berlin, Germany; isabel.schermuly@fu-berlin.de (I.I.S.); lucia.mastrototaro@ddz.de (L.M.); 2 American Institute for Goat Research, Langston University, Langston, OK 73050, USA; patra_amlan@yahoo.com; 3 Institute for Clinical Diabetology, German Diabetes Center, Leibniz Institute for Diabetes Research at Heinrich-Heine-University, Auf’m Hennekamp 65, 40225 Düsseldorf, Germany; 4 Animal Nutrition Services, Evonik Operations GmbH, Rodenbacher Chausee 4, 63457 Hanau-Wolfgang, Germany; andreas.lemme@evonik.com; 5 Institute of Animal Nutrition, Freie Universität Berlin, Königin-Luise-Straße 49, 14195 Berlin, Germany

**Keywords:** amino acid interaction, methionine supplementation, piglets, intestinal absorption, intestinal inflammation, Ussing chamber, angiotensin-converting enzyme II

## Abstract

**Background:** Methionine (Met) is a popular nutritional supplement in humans and animals. It is routinely supplemented to pigs as L-Met, DL-Met, or DL-2-hydroxy-4-(methylthio) butanoic acid (DL-HMTBA). **Methods:** We investigated the effect of these Met supplements on jejunal amino acid (AA) transport in male castrated Piétrain × Danbred pigs, also including a non-supplemented group. The mucosal-to-serosal flux of ten [^14^C]-labeled AAs (L-glutamine, glycine, L-leucine, L-lysine, L-Met, L-serine, L-threonine, L-tryptophan, L-tyrosine and L-valine) was investigated at two concentrations (50 µM and 5 mM). Inhibition of apical uptake by mucosal L-Met was also measured for these AAs. The intestinal expression of apical AA transporters, angiotensin-converting enzyme II and inflammation-related genes were compared with those of a previous study. **Results:** Except for tryptophan and lysine at 5 mM, all AA fluxes were Na^+^-dependent (*p* ≤ 0.05), and the uptake of most AAs, except glycine and lysine, was inhibited by L-Met (*p* < 0.001). A correlation network existed between Na^+^-dependent fluxes of most AAs (except tryptophan and partly glycine). We observed the upregulation of B^0^AT1 (*SLC6A19*) (*p* < 0.001), the downregulation of ATB^0,+^ (*SLC6A14*) (*p* < 0.001) and a lower expression of *CASP1*, *IL1β*, *IL8*, *TGFβ* and *TNFα* in the present vs. the previous study (*p* < 0.001). **Conclusions:** The correlating AAs likely share the same Na^+^-dependent transporter(s). A varying effect of the Met supplement type on AA transport in the two studies might be related to a different level of supplementation or a different inflammatory status of the small intestine.

## 1. Introduction

Methionine (Met) is an essential amino acid (AA) that must be acquired nutritionally [1]. It serves as an important precursor of the AAs taurine and cysteine (Cys) and, via the latter, of the crucial intracellular antioxidant glutathione [2]. S-Adenosylmethionine, a metabolite of Met, is a central methyl group donor [3]. The average requirement for Met in adults is between 13 and 15 mg/kg/d [4], an amount that can be increased by diseases like renal insufficiency [5]. While foods of animal origin are rich in Met, its contents are considerably lower in most plant-based protein sources such as legumes [6]. Thus, Met, especially L-Met, is an increasingly popular nutritional additive in humans. In growing pigs, Met is of an even greater importance because it is considered one of the production-limiting AAs [7]. Therefore, the dietary supplementation of Met in the form of L-Met, DL-Met or its hydroxy analog DL-2-hydroxy-4-(methylthio)-butanoic acid (DL-HMTBA) is common practice in pig feeding [7]. As the pig is an excellent model animal for human gastrointestinal physiology [8,9], the investigation of the effect of Met supplements in pigs can provide valuable information on its nutritional roles and effects in humans.

It has been shown before in pigs [10] and poultry [11] that AA supplementation can affect the expression of intestinal AA transporters. We have previously shown that DL-Met supplementation induced a Na^+^-dependent transport of L-Met in the jejunum of young pigs, whereas no Na^+^-dependent component of L-Met transport was observed after L-Met and DL-HMTBA supplementation [12]. Several Na^+^-dependent AA transporters are known to be expressed in the apical membrane of intestinal epithelial cells [2]. The main Na^+^-dependent apical Met transporter appears to be the broad neutral AA transporter B^0^AT1, which is a low-affinity but high-capacity transporter with affinity for a variety of neutral AAs [13]. It prefers AAs with large side chains (K*_m_* ~ 1–5 mM), while smaller AAs are transported with lower affinity [13]. Other Na^+^-dependent transporters are ATB^0,+^, ASCT2 and IMINO [14]. It was supposed that ATB^0,+^ is not expressed in pigs [15], which was refuted by later studies [16,17]. However, ATB^0,+^ mRNA expression was rather low in the small intestine, especially when compared to that in the large intestine [17]. It transports neutral and cationic AAs with a much higher affinity than B^0^AT1 (K*_m_* ~ 5–100 µM) [18]. The intestinal ASCT2 is an apical AA exchange system for neutral AAs [19], whereas the IMINO transporter is primarily a proline (Pro) transporter with an additional affinity for Met [20]. The Na^+^-independent b^0,+^AT preferably exchanges charged AAs and neutral AAs across the apical cell membrane [21]. Conversely, the methionine hydroxy analog HMTBA is not an AA and is taken up into the cell via H^+^-dependent monocarboxylate transporters [22].

As Met transporters not only accept Met but also a variety of other neutral and partly charged AAs [14], dietary Met supplementation might also have an impact on the intestinal uptake of other AAs. To elucidate the effects of DL-Met, L-Met or DL-HMTBA feeding on the intestinal uptake of different neutral AAs, we performed ex vivo flux and uptake studies in Ussing chambers. For comparison, L-lysine (Lys) was also included as a positively charged AA with high nutritional relevance. To conclude on the involved AA transporters, we investigated the Na^+^ dependency of transport at a low (50 µM) and high (5 mM) physiological mucosal AA concentration and the possible *cis*-inhibition of apical AA uptake by excess L-Met on the mucosal side. We additionally performed a correlation network analysis of Na^+^-dependent AA flux rates to explore which AAs might potentially use the same Na^+^-dependent AA transport mechanism. Consequently, the main objective of the present study was to evaluate the effects of diets supplemented with either DL-Met, L-Met, and DL-HMTBA or no Met supplement on the jejunal transport capacity for different neutral and charged AAs using an ex vivo Ussing chamber model. A second aim was to characterize the involved AA transporters and their dietary modulation in the porcine intestine using a flux rate correlation approach, an uptake experiment to investigate *cis*-inhibition of AA transport by L-Met and reverse-transcription quantitative PCR (RT-qPCR) of AA transporter genes, namely, B^0^AT1 (*SLC6A19*), ATB^0,+^ (*SLC6A14*), IMINO (*SLC6A20*) and ASCT2 (*SLC1A5*). We hypothesized that the dietary supplement, especially DL-Met, may increase the absorption of neutral and basic AAs and the expression of at least some of their transporter genes.

During the evaluation of the functional AA transport data, we observed different results to one of our earlier studies as we recorded no influence of the type of Met supplement on the Na^+^-dependent transport of L-Met. By contrast, we had previously observed Na^+^-dependent transport of L-Met only after supplementation with DL-Met [12].

We speculated that the latter discrepancy might be linked to a different inflammatory status as inflammation is not a rare occurrence in the postweaning period and may sometimes go unnoticed. It was shown earlier that intestinal inflammation can have profound effects on AA transport in the small intestine [23]. Particularly B^0^AT1 is linked to inflammation as it is associated with the angiotensin-converting enzyme II (ACE2) for functional expression at the cellular membrane [24]. The latter is not only involved in the regulatory renin–angiotensin–aldosterone system but also in inflammatory reactions [25] and was shown to be downregulated in intestinal inflammation [26]. This in turn may impede intestinal AA uptake, as B^0^AT1 is commonly considered the major apical uptake route for neutral AAs in the intestine [27]. To unravel the potential role of intestinal inflammation, we performed RT-qPCR of inflammation-related genes (caspase1 (*CASP1*), *NLRP3*, *IL1β*, *IL8*, *IL18*, *TNFα* and *TGFβ*) and *ACE2* and compared the results between the current and our earlier study. *NLRP3* and *CASP1* are parts of the so-called NLRP3 inflammasome, a protein complex that reacts with a wide variety of external and endogenous danger signals [28]. Its stimulation leads to the activation of the effectors *IL1β* and *IL18*, both of which mediate pro-inflammatory effects [28,29,30]. Likewise, *IL8* and *TNFα* are pro-inflammatory cytokines [31,32], while *TGFβ* has diverse regulatory effects on immune reactions [33]. The results of qRT-PCR analysis of inflammation-related genes were used together with the qRT-PCR results on AA transporter genes to elucidate the factors contributing to the differing functional results observed between the previous and the present study. We hypothesized that sub-clinical intestinal inflammation may have impeded the intestinal AA transport in the previous study.

## 2. Materials and Methods

### 2.1. Animal Ethics

All experiments involving pig handling and treatments were registered and approved by the local authorities (Reg. Nos. T 0264/15 and T 0264/20).

### 2.2. Animals and Diets

The current study was the conceptual continuation of a previous study (Trial A), which has been described in detail before [12]. Briefly, Trial A was performed using 27 male castrated Piétrain × Danbred pigs that were divided into three dietary treatment groups with nine animals each. The groups received a diet where deficiency in Met + Cys (basal content, 0.46%) was compensated by supplementation with either 0.21% L-Met, 0.21% DL-Met or 0.31% DL-HMTBA. As the level of Met supplementation was intentionally very high in this trial and comparison with Met deficiency was not an aim, a non-supplemented control group was not included. In the three feeding groups, the mucosal-to-serosal flux rates of D-Met and L-Met in the duodenum, jejunum and ileum [12], as well as the expression of Met transport proteins along the gastrointestinal tract, were studied [17].

The current functional study (Trial B and Trial C) comprised a total of 50 male castrated Piétrain × Danbred pigs to allow for a sample size of *n* = 10. The latter sample size was determined based on the results of Trial A to identify effects on Na^+^-dependent flux rates or uptakes among groups. Forty pigs were used to study the mucosal-to-serosal (ms) flux rates of ten different AAs in the mid-jejunum in Trial B. The pigs of Trial B were additionally used to study the expression of selected AA transport- and inflammation-related genes in the jejunum. Gene expression data of Trial B were compared to those of 27 jejunal samples harvested in Trial A [12]. The remaining 10 pigs were used in Trial C to measure the apical AA uptake in the mid-jejunum, applying the same AAs as those used in Trial B. Animals used in all trials were purchased from Asmussen Agro GmbH (Jessen, Germany) at an age of 10–15 weeks.

Trial B was performed in five consecutive runs with eight pigs per run. The eight pigs used in each run were randomly divided into four feeding groups that were housed in stainless steel frames of 1.9 m × 1.9 m at the Institute of Animal Nutrition of Freie Universität Berlin (Berlin, Germany). A control group received a moderately Met-restricted basal diet (0.57% Met + Cys; for diet composition, see Table 1). The diets of the other groups were of the same basal composition but were supplemented with either 0.15% L-Met, 0.15% DL-Met or 0.23% DL-HMTBA. The higher inclusion level of HMTBA was used to compensate for its lower bio-efficacy [34]. Allotment of groups to the four diets was blinded to the investigators during the trial, with diets being described as diets 1 to 4. After completion of the statistical analyses, all information concerning the identity of the four diets was released by the designer of the feed composition at EVONIK Operations GmbH.

For the uptake studies (Trial C), all 10 animals were kept in a 4 m × 2 m enclosure at the Institute of Pharmacology and Toxicology of Freie Universität Berlin. All pigs in Trial C received the diet supplemented with 0.15% DL-Met (Table 1 and Table 2) because only the DL-Met-supplemented diet had consistently elicited Na^+^-dependent flux rates of L-Met in Trials A and B.

All pigs had ad libitum access to feed and water.

### 2.3. Feed Analysis

Analyses of feed ingredients and diets were carried out at the laboratory of Evonik, Hanau-Wolfgang, Germany. Crude fiber, ether extract, ash, neutral detergent fiber, acid detergent fiber, starch, sugar and dry matter contents were measured by NIRS according to ISO 12099:2017 [35]. Crude protein and AA were analyzed by applying the method of Llames and Fontaine [36]. Nitrogen was determined using a Leco FP-2000 Nitrogen Analyzer (Leco Corporation, St. Joseph, MI, USA).

### 2.4. Tissue Sampling

After at least 10 days on the experimental diet, one animal per day was randomly chosen and killed. The method of euthanasia and tissue sampling was described previously [12,37]. In short, the animals were sedated by intramuscular injection with 40 mg/kg ketamine (Ursotamin 100 mg/mL, Serumwerk Bernburg, Bernburg, Germany) and 8 mg/kg azaperone (Stresnil, 40 mg/mL, Elanco Animal Health, Bad Homburg, Germany). After reaching a state of deep sedation, pigs were euthanized by intracardial injection of embutramide/tetrainhydrochloride/mebezoniumchloride (T61, Intervet Deutschland GmbH, Unterschleißheim, Germany). From the opened abdominal cavity, the jejunal tissue was harvested and cut open longitudinally. Afterwards, the tissue was thoroughly rinsed in heated (37 °C) buffered solution (GB) containing (in mM) 135 NaCl, 1 MgCl_2_, 1.8 CaCl_2_, 10 HEPES, 5 *N*-methyl-D-glucamine (NMDG^+^), 0.5 KH_2_PO_4_, 2.5 K_2_HPO_4_, 10 glucose and an AA mix composed of 23 AAs (pH 7.4, 288 mosmol/l) [12]. For transport, enrofloxacin was added to the GB (27.8 µM). During transport to the lab, the jejunal tissue was kept in heated (37 °C) GB and continuously gassed with O_2_.

Samples for RT-qPCR were cleaned with cold GB and stored at −20 °C in RNA*later*^®^ (Sigma Aldrich, St. Louis, MO, USA).

### 2.5. Ussing Chamber—Amino Acid Flux (Trial B)

The Ussing chamber method was described in detail previously [12,37]. In the present study, the jejunal tissue was bathed in 15 mL of buffered solution on each side. The serosal side was incubated with GB, whereas the mucosal side received a glucose-free buffered solution (MB) with a composition like GB except that glucose was iso-osmotically replaced by mannitol and that it did not contain the AA mix [12]. To evaluate the Na^+^ dependency of AA transport, AA flux rates were additionally measured in the absence of mucosal Na^+^, using NMDG^+^ as an equimolar substitute of Na^+^.

The ms flux rates of ten partially [^14^C]-labeled AAs were investigated using a 9.25 kBq radioactive label on the mucosal side of each chamber. Amino acids included L-glutamine (Gln), glycine (Gly), L-leucine (Leu), Lys, L-Met, L-serine (Ser), L-threonine (Thr), L-tryptophan (Trp), L-tyrosine (Tyr) and L-valine (Val) (all from Hartmann Analytic GmbH, Braunschweig, Germany). Final mucosal concentrations of 50 µM or 5 mM of the respective AAs were reached by the simultaneous addition of the respective non-labeled AAs. Flux measurements of all aforementioned AAs under different conditions (mucosal AA concentration/presence of mucosal Na^+^) were conducted for each animal in the study, resulting in the utilization of 40 chambers per animal. Labeled and non-labeled AAs were added to short-circuited jejunal epithelia ~20 min after mounting at t = 0 min. Duplicate mucosal samples (2 × 100 µL) were taken immediately thereafter. At t = 30 min, the first duplicate samples (2 × 600 µL; P0) were taken from the serosal side. Further duplicate serosal samples (2 × 600 µL) were taken at t = 75 min (P1) and 120 min (P2). After each serosal sampling, 1.2 mL of fresh GB solution was added to the serosal solution to keep the hydrostatic pressure stable. A second duplicate mucosal sample (2 × 100 µL) was taken at t = 130 min. After the last flux period, the viability of the tissues was validated using 8 mM of theophylline bilaterally, which induced a short-circuit response in all tissues. Mucosal samples were filled up to 600 µL with GB before 3 mL of Rotiscint^®^ eco plus (Carl Roth GmbH + Co. KG, Karlsruhe, Germany) was added to each mucosal and serosal sample. Samples were then put on a rotating agitator for 10 min. The radioactivity of samples was measured using a liquid scintillation β-counter (TRI-CARB 4910TR, Perkin-Elmer, Rodgau, Germany). The disintegrations per minute (DPM) were counted with the protocol ^14^C DPM for 2 min (0–156 keV). Unidirectional ms flux rates were calculated by the appearance of radioactive tracers on the serosal side with the equation of Schultz and Zalusky [38]. The arithmetic mean values of flux rates from P1 and P2 were subjected to statistical analyses.

### 2.6. Ussing Chamber—Amino Acid Uptake (Trial C)

Uptake experiments were performed in the presence and in the absence of mucosal Na^+^, using the same bathing solutions like in Trial B. The same partially [^14^C]-labeled AAs were used (18.5 kBq per chamber), except L-Met, at a final AA concentration of 50 µM. For each AA, one pair of chambers with Na^+^-containing and Na^+^-free mucosal solution, respectively, received mucosal L-Met (5 mM) to test for the possible *cis*-inhibition of AA uptake, whereas another chamber pair from the same pig did not receive L-Met additions.

At the start of experiment, all chambers were incubated with GB and MB on their serosal and mucosal sides, respectively. Before uptake measurements, the mucosal solution was completely replaced with either Na^+^-containing or Na^+^-free MB as appropriate. To ensure the removal of all Na^+^ in chambers receiving Na^+^-free MB, an additional wash with 12 mL Na^+^-free MB was included in the replacement procedure. At 2 min and 50 s after replacement of the mucosal solution, 5 mM unlabeled L-Met was added to the mucosal solution of chambers intended for assessment of *cis*-inhibition. The partially radiolabeled AA was added to the mucosal side of the Ussing chamber at 3 min after replacement of the mucosal solution. At 20 s after isotope addition, a mucosal sample (2 × 100 µL) was taken. At exactly 1 min after isotope addition, the chamber was rinsed three times with 12 mL of ice-cold Na^+^-free MB. The tissue was immediately removed from the chamber and a circular punch sample was taken from the middle of the tissue (2.83 cm^2^). Each tissue sample was placed in 5 mL of lysis solution (0.2 M NaOH + 0.25% SDS) and vortexed for 30 s. Samples were then placed on a rotating agitator for 4 min. After another 30 s of vortexing, the remaining tissue was removed from the vials and the samples were centrifuged at 5000× *g* and 4 °C for 20 min. Two samples (600 µL) were drawn from the lysate. The radioactivity of samples was determined as described above. The apical AA uptake (in nmol·cm^−2^·min^−1^) was calculated using the following equation:

(1) $$U=\frac{V_{t}\times C_{n}\times \frac{{dpm}_{n}\times V_{l}}{V_{c}}}{\frac{{dpm}_{h}\times V_{t}}{V_{h}}\times A}$$

where U, uptake [nmol·cm^−2^·min^−1^]; C_n_, AA concentration in mucosal solution [M]; V_t_, total volume of mucosal solution [L]; V***_l_***, volume used for tissue lysis [L]; V_c_, volume of lysis solution used for radioactivity counting [L]; V_h_, volume of hot sample from mucosal solution [L]; dpm_n_, radioactivity counted in aliquot of lysis solution [DPM]; dpm_h_, radioactivity counted in hot sample [DPM]; and A, surface area of lysed tissue [cm^2^].

### 2.7. Reverse-Transcription Quantitative PCR

Reverse-transcription quantitative PCR analysis was performed for the following genes: *SLC6A19* (B^0^AT1), *SLC6A14* (ATB^0,+^), *SLC6A20* (IMINO), *SLC1A5* (ASCT2), *ACE2* (angiotensin-converting enzyme II), *CASP1* (caspase 1), *NLRP3* (NLR family pyrin domain containing 3), *IL1β*, *IL8*, *IL18*, *TNFα* (tumor necrosis factor α) and *TGFβ* (transforming growth factor β). Primers were purchased from Eurofins (MWG Operon, Ebersberg, Germany). Analysis of transporter genes and *ACE2* was performed with iTaq^®^ Universal Probes Supermix (Bio-Rad, Hercules, CA, USA), and analysis of inflammation markers was performed with iQ^TM^ SYBR Green Supermix^®^ Kit (BioRad, Hercules, CA, USA). Methodological details for RNA isolation, RNA integrity checks, cDNA production and running method were already published [17,39]. The primer sequences used for the analysis of Met transporters and inflammatory markers are shown in Table 3. Glycerinaldehyde-3-phosphate dehydrogenase (*GAPDH*) and 14-3-3 protein zeta/delta (*YWHAZ*) were used for normalization as unregulated housekeeping genes. Both housekeeping genes were validated using GeNorm [40]. A pooled sample composed of all used cDNA samples was present on each plate and was used as inter-run-calibrator (IRC) whenever multiple plates were run for a given gene (i.e., inflammation-related genes). Thresholds were automatically calculated by the ViiA7 1.2 software and aligned with the IRC. The data were normalized to the two housekeeping genes and the pooled sample using the 2^−∆∆Ct^ method [41].

### 2.8. Statistical Analyses

**Figure 1:**
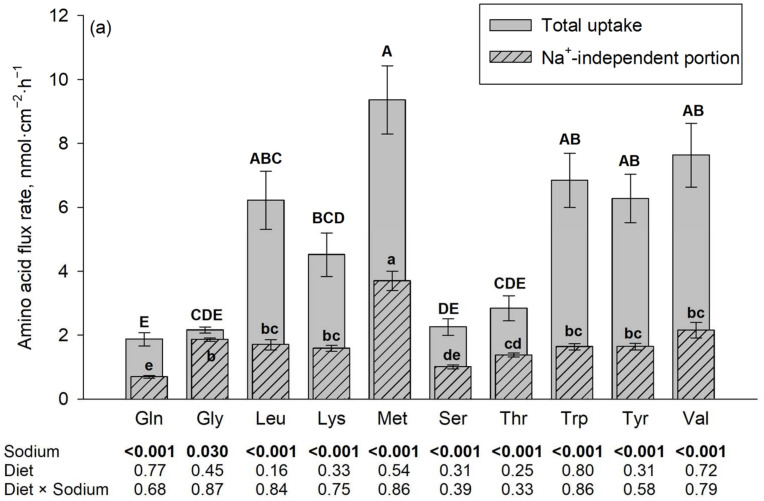

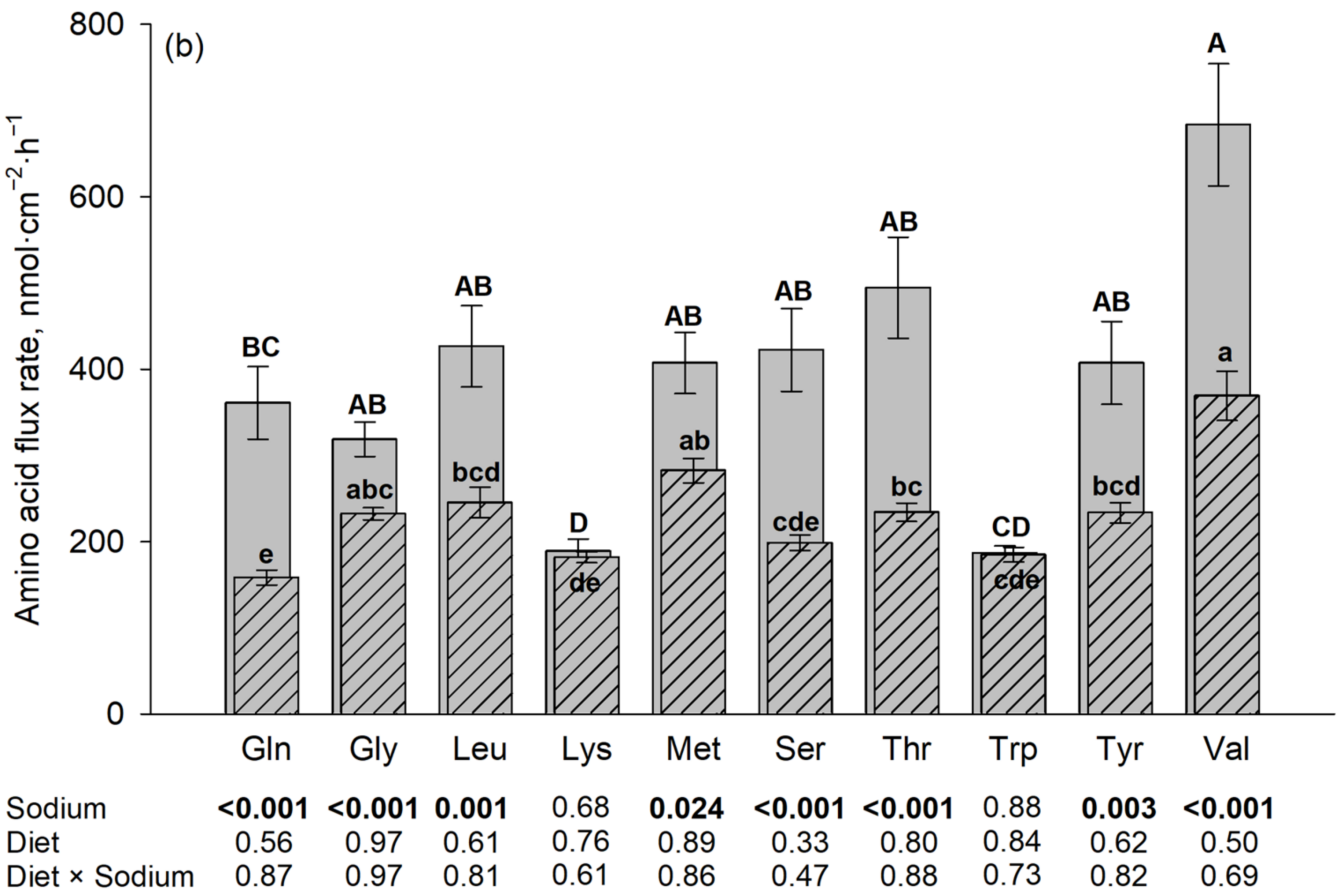
Jejunal amino acid flux rates in the presence and absence of Na^+^, the latter using *N*-methyl-D-glucamine (NMDG^+^) as a Na^+^ replacement. The flux rate in the presence of Na^+^ is the total flux rate and represented by each plain gray column in the back. The hatched column in the front represents the Na^+^-independent portion and the non-hatched part above each hatched column represents the Na^+^-dependent portion of the total amino acid flux rate. Mucosal amino acid concentration was 50 µM (**a**) or 5 mM (**b**). Results are given as means ± SEM for the factor sodium of *n* = 7–10 pigs. Respective *p*-values for the influence of sodium and diet are indicated below each amino acid. ^A–E^ Different capital letters above columns indicate differences in total flux rates, and ^a–e^ different small letters indicate differences in their Na^+^-independent portions among all tested amino acids. Gln, L-glutamine; Gly, glycine; Leu, L-leucine; Lys, L-lysine; Met, L-methionine; Ser, L-serine; Thr, L-threonine; Trp, L-tryptophan; Tyr, L-tyrosine; and Val, L-valine.

With the exception of correlation network analysis, statistical analyses and production of graphs were executed with SigmaPlot 11.0 (Systat Software, GmbH, Erkrath, Germany). The experimental unit was the individual pig. Statistical analysis of data started with a Shapiro–Wilk’s test for normal distribution. Data that were not normally distributed were log_10_-transformed before group comparisons to reach normal distribution. For group comparisons of factorial data, two-way ANOVA was applied with factors such as diet (L-Met, DL-Met, DL-HMTBA and control) and sodium (Na^+^ and NMDG^+^) for flux rates, factors such as sodium (Na^+^ and NMDG^+^) and *cis*-inhibition (0 mM Met and 5 mM Met) for uptakes, and factors such as diet (L-Met, DL-Met, and DL-HMTBA) and trial (Trial A and Trial B) for molecular biological analyses. All three analyses were followed by a Student–Newman–Keul’s post hoc test. The comparisons between the flux rates of different AAs in Figure 1 were made separately for total flux rates and Na^+^-independent flux rates by using ANOVA on ranks followed by Dunn’s test. Differences of *p* ≤ 0.05 were considered significant; 0.05 < *p* ≤ 0.10 was considered a trend. Because only one observation was made for each given variable per pig, the number of observations always equals the number of experimental animals used, which is given as *n*. Data removal was based solely on the identification of technical errors during the experiments.

The Na^+^-dependent flux rates used for correlation network analysis were calculated by subtracting the flux rates in the absence of Na^+^ from the flux rates in the presence of Na^+^. Pearson correlation coefficients (*r*) between AA flux rates were calculated using SAS (9.4 User’s Guide; SAS Inst. Inc. Cary, NC, USA, 2011), and correlation networks were constructed for flux rates with significant (*p* ≤ 0.05) correlation coefficients using Cytoscape (version 3.6.1) [44].

## 3. Results

### 3.1. Ussing Chamber—Amino Acid Flux Rates (Trial B)

The results on flux rates of the 10 different amino acids in the presence vs. absence of Na^+^ are summarized in Figure 1. The presence of Na^+^ increased the flux rates of almost all investigated amino acids (*p* ≤ 0.05). The only exceptions were Trp and Lys at a 5 mM mucosal concentration. The diet, as well as the interaction between diet and sodium, had no significant effect on the flux rates of any of the tested AAs at both AA concentrations (*p* > 0.05).

At a mucosal concentration of 50 µM and in the presence of Na^+^, the highest flux rates of all tested AA were observed for L-Met, followed by Val, Trp, Tyr, Leu and Lys, with lowest flux rates for Thr, Ser, Gly and finally Gln. The Na^+^-independent portions of flux rates were also the highest for L-Met and the lowest for Gln, Ser and to some extent Thr, with intermediate values for the remaining AAs. For Gly, the difference between total flux rate and Na^+^-independent flux rate (representing the Na^+^-dependent flux rate) was negligible (despite being statistically significant).

At a mucosal concentration of 5 mM and in the presence of Na^+^, the highest flux rates were observed for Val, followed by Thr, Ser, Leu, L-Met, Tyr, Gln, and Gly with lowest flux rates observed for Trp and Lys. The latter two AAs were also the AAs where no difference existed between total and Na^+^-independent flux rates. The Na^+^-independent portions of flux rates at the 5 mM mucosal concentration were also the highest for Val followed by L-Met and the lowest for Trp and Lys together with Gln and Ser, with intermediate values for the remaining AAs.

### 3.2. Correlation Network Analysis

**Figure 2:**
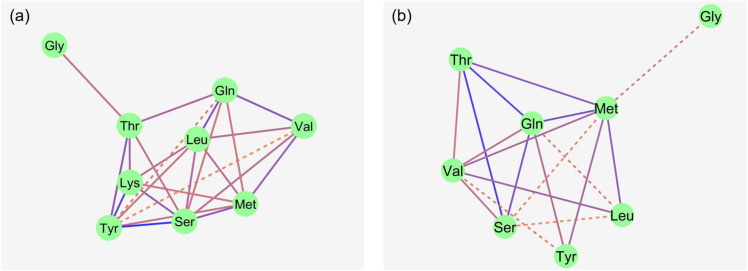
Correlation network of Na^+^-dependent AA flux rates in the porcine middle jejunum ((**a**): 50 µM AA concentration; (**b**): 5 mM AA concentration). *n* = 32–40 pigs. Dotted lines: 0.01 < *p* ≤ 0.05; solid lines: *p* ≤ 0.01. Color of lines: correlation decreases from blue to red. For abbreviations and individual correlation coefficients, see Table 4.

The Na^+^-dependent portion of flux rate was calculated for each AA as the difference between the flux rates in the presence and absence of Na^+^ (equivalent to the non-hatched portion extending above the hatched portion (shown in Figure 2)). The Na^+^-dependent flux rates represent transport via Na^+^-dependent AA transporters. To elucidate whether the tested AAs use the same Na^+^-dependent AA transporters, a correlation network analysis was performed.

At 50 µM, flux rates of non-polar neutral AAs like L-Met, Val and Leu showed several correlations with each other. Strong correlations were also observed between the flux rates of polar AA like Ser, Thr, Tyr and Gln. The charged AA Lys showed multiple correlations with the polar AAs (Ser, Thr and Tyr), as well as Leu and L-Met. Gly flux rates correlated with only Thr flux rates. No correlation was observed between Trp and any other AA flux rate.

At the 5 mM mucosal AA concentration, Lys and Trp were removed from the model because these AAs did not show any significant Na^+^-dependent flux rates. Correlations between the remaining AAs at 5 mM were partly similar to those at 50 µM of AAs. The most striking difference was that Ser and Thr flux rates lost their strong relationship to the Tyr flux rates observed at 50 µM, while Tyr flux rates now showed intense relationships to the flux rates of the large non-polar AAs L-Met and Gln. Gly was only correlated, as a trend, to L-Met. Individual correlation coefficients are shown in Table 4.

### 3.3. Ussing Chamber—Amino Acid Uptake (Trial C)

**Figure 3:**
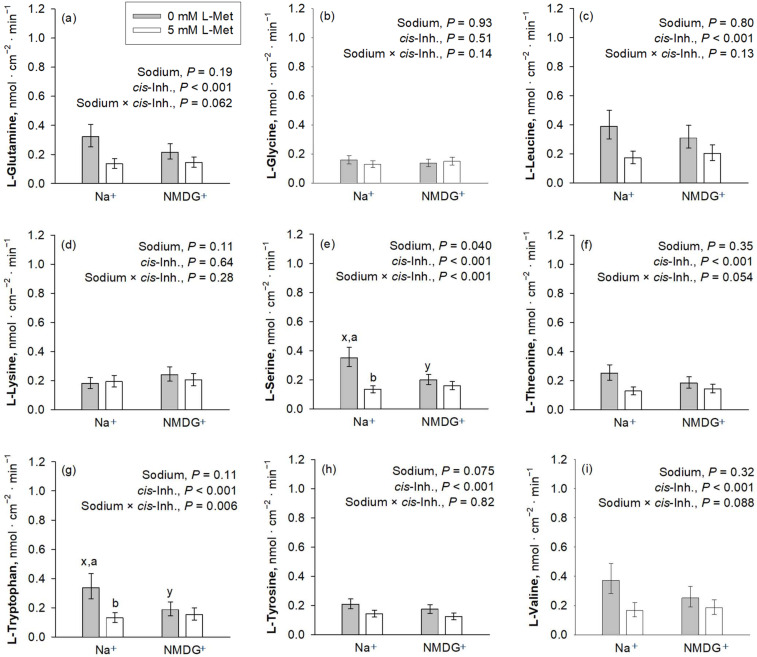
Apical uptakes of the amino acids Gln, Gly, Leu, Lys, Ser, Thr, Trp, Tyr and Val (**a**–**i**) at a concentration of 50 µM. Uptakes were measured in the presence or absence of mucosal Na^+^ and the presence or absence of 5 mM mucosal L-Met, with the latter representing the factor *cis*-inhibition of amino acid uptake. Data are presented as least square mean ± confidence interval of *n* = 8–10 pigs. Data were compared with a two-way ANOVA and a post hoc Student–Newman–Keul’s test. Factor *p*-values for sodium and *cis*-inhibition by L-Met, as well as their interaction, are cited in each graph. ^x,y^ depict differences within the factor sodium, whereas ^a,b^ indicate differences within the factor *cis*-inhibition in the same graph panel (*p* ≤ 0.05). Inh., inhibition; NMDG^+^, *N*-methyl-D-glucamine.

*cis*-Inhibition of the AA uptake by the mucosal presence of L-Met was observed for almost all investigated AAs, except Gly and Lys (Figure 3). The uptakes of Gly and Lys were further not influenced by the presence of Na^+^ (*p* > 0.05). Among those AAs where uptake was inhibited by L-Met, a significant sodium × *cis*-inhibition effect was present for Trp and Ser (*p* ≤ 0.05), while Val, Gln and Thr showed a trend for a sodium × *cis*-inhibition effect (0.05 < *p* ≤ 0.10). The *p*-value for sodium × *cis*-inhibition was 0.13 for Leu, whereas it was 0.82 for Tyr, suggesting that, at least for the latter, inhibition by L-Met was present in both Na^+^-containing and Na^+^-free mucosal solution.

### 3.4. Quantitative Real-Time PCR—Transporters

**Figure 4:**
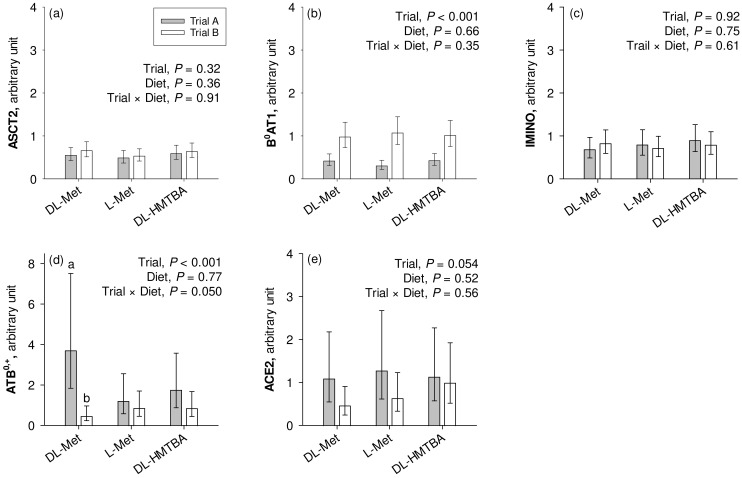
Calibrated normalized relative quantity of gene expression of apical Na^+^-dependent amino acid transporters ASCT2, B^0^AT1, IMINO, and ATB^0,+^ and the angiotensin-converting enzyme II (*ACE2*) (**a**–**e**) in the jejunum of pigs of two different trials, each using three similar diets. Results are given as least square mean ± confidence interval of *n* = 8–10 pigs. Results were analyzed by two-way ANOVA with the factors trial and diet. Factor main effects and their interactions are cited in each graph. ^a,b^ Different small letters indicate differences within the factor diet in the same graph panel (*p* ≤ 0.05). DL-HMTBA, DL-2-hydroxy-4-methylthiobutanoic acid.

The mRNA expression of B^0^AT1 was significantly higher in the pigs from the current Trial B compared to the pigs from the previous Trial A (*p* < 0.001; Figure 4). On the other hand, pigs from Trial A had significantly higher ATB^0,+^ expression than the animals from Trial B (*p* < 0.001), which specifically related to animals from the group supplemented with dietary DL-Met as evidenced by a significant trial × diet interaction effect (*p* = 0.050). *ACE2* tended to show greater expression in pigs used in Trial A than in Trial B (0.05 < *p* ≤ 0.10). ASCT2 and IMINO showed similar mRNA expression levels in Trials A and B (*p* > 0.10). Except for the mentioned trial × diet interaction for ATB^0,+^, the factor diet had no influence on transporter mRNA expression (*p* > 0.05).

### 3.5. Quantitative Real-Time PCR—Inflammatory Markers

**Figure 5:**
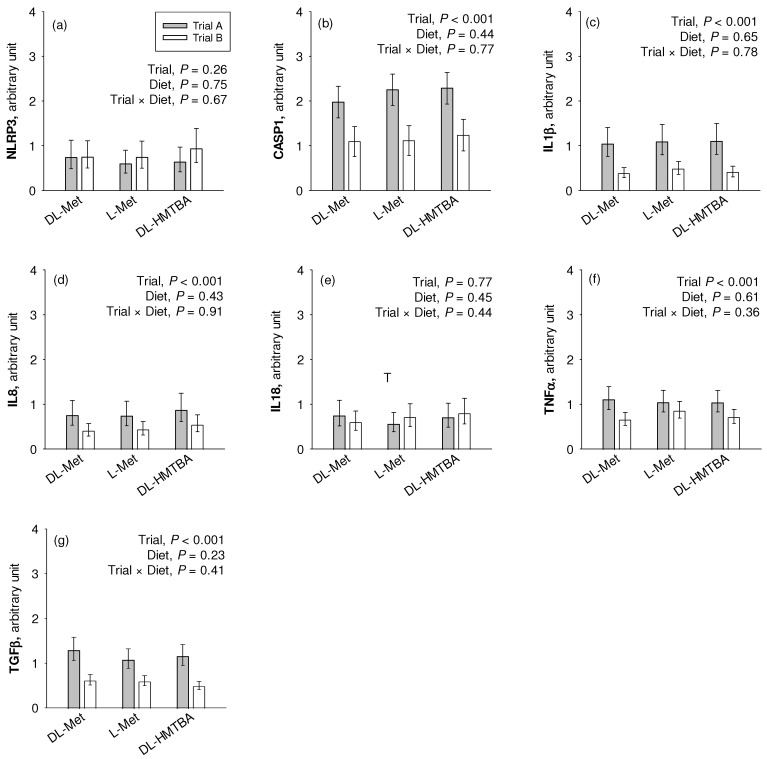
Comparative relative gene expression of the inflammation-related markers NLR family pyrin domain containing (NLRP)3 (**a**), caspase (CASP)1 (**b**), interleukin (IL)1β (**c**), IL8 (**d**), IL18 (**e**), tumor necrosis factor (TNF)α (**f**) and transforming growth factor (TGF)β (**g**) in the jejunum of pigs of two different trials fed three different diets. Results are given as least square mean ± confidence interval of *n* = 9–10 pigs. Results were analyzed by two-way ANOVA with the factor trial and diet. Factor main effects and their interaction are depicted in each graph. DL-HMTBA, DL-2-hydroxy-4-methylthiobutanoic acid.

We observed a significantly higher mRNA expression of *CASP1*, *IL1β*, *IL8*, *TGFβ,* and *TNFα* (*p* < 0.001) in the pigs of Trial A compared to those of Trial B (Figure 5). *NLRP3* and *IL18* were not affected by the factor trial. Diet effects or trial × diet interaction effects were not observed for any gene (*p* > 0.05).

## 4. Discussion

Pigs are considered a first-choice model for human gastrointestinal physiology and pathophysiology. The present study is a continuation of our investigations on amino acid absorption in the small intestine of this model species. The main focus was on the intestinal transport of the limiting AA L-Met and the effects of three widely used Met supplements (DL-Met, L-Met and DL-HMTBA) on this transport. In a previous study, we showed that supplementation of DL-Met had stimulating effects on absorptive L-Met flux rates in three small intestinal segments (the duodenum, jejunum and ileum), with Na^+^-dependent transport present only in the jejunum of DL-Met-supplemented piglets and only at a mucosal concentration of 50 µM L-Met [12]. The original intention of the present study was to test whether dietary supplementation with DL-Met may equally promote the intestinal absorption of other neutral AAs (Gln, Gly, Leu, L-Met, Ser, Thr, Trp, Tyr and Val) and possibly also the absorption of the other main limiting AA Lys with which Met can share transport via the ATB^0,+^ and b^0,+^AT/rBAT transporters [18,45]. The concentrations of AAs (50 µM and 5 mM) were chosen to cover the relevant range based on the finding that luminal free AA concentrations in the lower millimolar range can be expected in the postprandial state in pigs [46]. Correlation network analyses were intended to elucidate possible relationships between the absorptive flux rates of the different AAs to conclude on common transport pathways, i.e., on commonly used AA transporters.

One striking finding was that L-Met (as well as the other investigated AA) was transported to a significant part in a Na^+^-dependent manner with no influence of experimental diet on absorptive flux rates. For L-Met (and most other investigated AAs except Lys and Trp), the Na^+^ dependence of absorption was not only observed at a low (50 µM) but also at a high luminal concentration (5 mM) across diets. These findings are different to our previous study and reinforce that Na^+^-dependent transport is an important contributor to AA absorption at low and partly at high luminal concentrations [14].

From a quantitative point of view, L-Met had the highest absorptive flux rate amongst all tested AAs at 50 µM, both in the presence and in the absence of Na^+^. This is in agreement with a previous postulate that Met is the most effectively absorbed AA in the gastrointestinal tract [47]. The other AA effectively absorbed at 50 µM included all large neutral AAs (Trp, Tyr and Leu) except Gln, as well as Val and partly Lys. A preferential absorption of Met in the presence of Na^+^ is classically attributed to the B^0^AT1 transporter that is also known as the “methionine-preferring system” [2,14]. L-Met also had the highest absorptive flux rate in the absence of Na^+^. As L-Met is one of the largest AA tested, this very high Na^+^-independent flux rate cannot be attributed to passive diffusion across the paracellular space (which is size-restricted [48]) but must be mediated, at least to a major part, by a Na^+^-independent transporter. The Na^+^-independent transporter responsible for Na^+^-independent L-Met flux is most likely b^0,+^AT/rBAT, which was previously identified as a main Met influx carrier in human Caco2 cells [49]. Proceeding from an involvement of b^0,+^AT/rBAT in the Na^+^-independent absorption of L-Met and other basic and neutral AAs including Gln, it is surprising that absorptive flux rates of Gln were so low in the absence of Na^+^. As the flux rates of Gln were also the lowest in the presence of Na^+^ at the low concentration of 50 µM, one possible explanation could be Gln metabolism, given the fact that Gln is a preferred substrate for energy metabolism of intestinal epithelial cells [50]. As such, the comparatively low flux rates of Gln in the absence and in the presence of Na^+^ could be related to the metabolism of Gln with the subsequent fixation of Gln in, e.g., glutathione after partial metabolism or escape of the [^14^C]-radiolabel as ^14^CO_2_ after complete metabolism. Indeed, Gln is intensely metabolized in the intestinal epithelium, with CO_2_ being a major metabolite [51]. Other AAs may also be metabolized in the intestinal epithelial cells. Met, for example, is also intensively metabolized in the epithelium; however, the emerging metabolites of Met metabolism (especially homocysteine) are considered to leave the epithelium on the serosal side [2] and would thus be included in the serosally appearing radiolabel used for the calculation of L-Met flux rates.

An increase in AA concentration from 50 µM to 5 mM (i.e., by a factor of 100) led to an over-proportional increase in the total flux rates of Gln, Ser and Thr by a factor of ~200. In the case of Gln, this could possibly be interpreted by a saturation of metabolism at higher Gln concentrations, leading to higher flux rates of non-metabolized Gln. However, as the Na^+^-dependent flux rates of Gln, Ser and Thr were amongst the most strongly correlating flux rates especially at 5 mM concentration, this could also suggest the induction/stimulation of a common Na^+^-dependent transport system for these three (and possibly other) AAs. High concentrations of Gln were previously shown to increase the abundance of the neutral AA transporters B^0^AT1 and ASCT2 in the intestinal brush border membrane within 3 min [52]. A later study on the murine intestine disputed the acute dietary regulation of B^0^AT1 [53]. Moreover, the substrates Gln, Ser and Thr are rather characteristic of ASCT2 [19]. Especially Ser and Thr have a lower affinity for other transporters like B^0^AT1 and ATB^0,+^ that preferentially accept AAs with larger side chains [14]. These considerations allow preference for the postulate of ASCT2 induction at higher AA concentrations. Conversely, a low functional activity of ASCT2 at a low AA concentration could explain the comparatively low Na^+^-dependent flux rates for Thr, Ser, Gln and finally Gly. The latter AA, Gly, is accepted with a lower affinity by ASCT2 [19], and a common transport of part of Gly via ASCT2 might potentially be deduced from its flux correlation to, at least, Thr at a 50 µM AA concentration.

As mentioned earlier, flux rates of Lys and Trp were not affected by the presence of Na^+^ at a mucosal concentration of 5 mM. In the uptake experiments, Trp uptake was strongly inhibited by mucosal L-Met. This *cis*-inhibitory effect was dependent on the presence of Na^+^. Consequently, L-Met and Trp probably shared at least one common apical transport system in our trial. It is well accepted that the majority of Trp is absorbed by the apical Na^+^-dependent neutral AA transporter B^0^AT1 [54]. This is supported by the pronounced intestinal and renal wasting of Trp in Hartnup disorder, a hereditary disease affecting the B^0^AT1 transporter [54]. The lack of Na^+^-dependent transport of Trp at a 5 mM concentration might either indicate the saturation of the transporter or that an excess of Trp might impair Trp transport via B^0^AT1 to prevent excessive L-Trp uptake. Indeed, it was shown that high AA concentrations are able to inhibit the expression of B^0^AT1 in a renal cell model [55]. In hamster intestinal sacs, it was described that Trp transport decreased at Trp concentrations > 5 mM and approached zero at 20 mM [56]. Similar results were obtained later by another group at a concentration of 10 mM Trp [57]. Some of these studies [55,56] proposed that a high Trp concentration damaged the tissue. In support of this proposal, it was shown in the hamster small intestinal epithelium that mucosal Trp concentrations exceeding 1 mM disrupted the tight junction structure, altered the cytoskeleton and increased the paracellular passage of Trp [58]. We did not measure paracellular permeability in our experiment. However, the completely equal and comparatively low flux rates of Trp in the presence and absence of Na^+^ at 5 mM strongly argue against paracellular damage. Moreover, we performed vitality tests at the end of each experiment; these vitality tests did also not indicate epithelial damage. Hence, our results argue in favor of a specific inhibitory effect of high Trp concentrations on Na^+^-dependent Trp absorption with no indication that this is linked to epithelial/paracellular damage. An autoregulation of Trp transport by Trp concentration could further explain that Trp flux rates did not correlate with flux rates of any other B^0^AT1 substrate AA even at a 50 µM concentration. This may be seen in the context of the classification of Trp as a “functional AA” [59]. Functional AAs possess regulatory properties on key metabolic pathways beyond their basic function [59]. Tryptophan, in particular, is the precursor of functional substances like kynurenins, serotonin, and others [60]. Likewise, arginine, Cys, Gln, Leu and Pro were classified as functional AAs previously [59], implying that these AAs may have a regulatory impact on their own transport. It is important to note that essential AAs not only possess regulatory properties, such as Pro and its derivatives that can regulate gene expression [59,61], but also they can protect from oxidative stress [62]. Additionally, Pro-rich proteins can bind anti-nutritive dietary components like tannins [63,64].

Considering that Trp is mainly taken up by B^0^AT1, the absent correlation of Na^+^-dependent Trp flux rates with the Na^+^-dependent flux rates of other neutral AAs, therefore, does not question the involvement of B^0^AT1 in the AA transport of the present study. The B^0^AT1 transporter has an acknowledged central role for neutral AA transport in the small intestine with a preference for large AAs [14]. Our correlation analyses validated correlations among almost all neutral AA and uptake studies revealed that the majority of investigated AAs (except Lys and Gly) were or tended to be inhibited by a surplus of L-Met (5 mM). The latter indicates that these AAs are transported by transporter(s) that also accept L-Met. The B^0^AT1 transporter is known as the “methionine-preferring system” [2,14]; therefore, it seems fair to propose that a major part of neutral AAs was transported via B^0^AT1 in our study.

Lys is a cationic AA and, thereby, its transport pathways are limited compared to those of neutral AAs. In epithelial cells, Na^+^-dependent and Na^+^-independent apical uptake of Lys are thought to be mediated by ATB^0,+^ and b^0,+^AT/rBAT, respectively, while basolateral efflux is facilitated via y^+^LAT1/4F2hc and y^+^LAT2/4F2hc [14]. The presence of ATB^0,+^ was previously disputed for the porcine small intestine [15] but acknowledged in later reports [16,17]. Furthermore, the rather low expression of ATB^0,+^ was previously used to suggest that there could be a transporter similar to ATB^0,+^ that mediates Na^+^-dependent absorption of Lys and other cationic AAs in the small intestine [14]. Our experiments are at least compatible with a functional presence of ATB^0,+^ or an ATB^0,+^-like transporter in the porcine jejunum. As expected for a high-affinity transporter (K*_m_* of ATB^0,+^ for Lys = 100 µM [18]), a significant Na^+^-dependent transport of Lys was observed at 50 µM Lys concentration but disappeared at 5 mM Lys concentration, with the latter being typical for transporter saturation. The flux rates of Lys at 50 µM concentration were further correlated to the flux rates of several neutral AAs, namely, L-Met, Ser, Thr and Tyr, which are among the known substrates of ATB^0,+^ [18]. Contrary to the flux experiments, we did not observe Na^+^ dependency in the 1 min uptake period and no inhibition by L-Met. The reason for this remains unknown but may be attributable to a delayed response of ATB^0,+^ transport to Lys substrate availability.

Taken together, the present study provided indication for the functional presence of at least three Na^+^-dependent transport systems in the jejunum of piglets independent of pre-feeding with or without one of the available dietary Met supplements (L-Met, DL-Met and DL-HMTBA). The transporters were B^0^AT1, ASCT2 and ATB^0,+^ (or alike), of which the latter was probably not active in the short-term uptake experiments. These results are largely compatible with textbook knowledge and thereby underline the suitability of the pig as a model for research in human nutrition. However, the results differed greatly from our previous study where we could not identify any Na^+^-dependent transport of Met after dietary supplementation with L-Met or DL-HMTBA. In that previous study, we observed Na^+^-dependent transport of L-Met only after pre-feeding with DL-Met and attributed this to a likely induction/preservation of ASCT2 function [12].

When searching for an explanation for the divergent results, the most obvious difference between the current and the previous studies is dietary composition. While the total Met + Cys supply was similar in both studies, the Met + Cys content of the basal diet was considerably higher in the present vs. the previous study (0.57% vs. 0.46%) with the consequence that the level of dietary Met supplementation was much lower in the present vs. the previous study (0.15% vs. 0.21%). This would lead to the speculation that the very low protein-bound Met + Cys level may compromise Na^+^-dependent absorption of Met and other neutral AAs in the jejunum of piglets, which can be partly prevented by DL-Met supplements but not by L-Met or DL-HMTBA supplements. In agreement with this speculation, Jando et al. showed that uptake of L-isoleucine into proximal jejunal rings of rats increased after 7 d on a diet high in true protein but not after 7 d on a normal-protein diet supplemented with crystalline AAs to the same crude protein level as the high-protein diet [53].

The study by Jando et al. had also indicated that B^0^AT1 transport function may be divergent from B^0^AT1 protein levels detectable in the intestinal brush border membrane, which was further divergent from B^0^AT1 mRNA levels [53]. Nevertheless, we compared the mRNA expression of selected Na^+^-dependent apical transporters, B^0^AT1, ASCT2, ATB^0,+^ and IMINO, with an attempt to substantiate the proposed presence of B^0^AT1 in the present experiment as opposed to the previous study. Indeed, the mRNA expression of only B^0^AT1 was significantly upregulated in pigs of the present study compared to the previous one. This may support the conclusion that B^0^AT1 could have made the difference between the two studies, i.e., the functional presence of B^0^AT1 likely contributed to Na^+^-dependent absorptive flux rates of L-Met and other neutral AAs across groups in the present study.

Apart from dietary regulation, B^0^AT1 expression and function may also be compromised by intestinal inflammation [65,66]. Our experiments revealed a higher mRNA expression of several pro-inflammatory cytokines in animals of the previous Trial A compared to animals of the current Trial B. Besides *TNFα* and *TGFβ*, we also observed the upregulation of *CASP1*, *IL1β* and *IL8* in pigs from Trial A, all of which mediate pro-inflammatory reactions [32,67]. Therefore, a lower inflammatory status may be another reason for the presence of Na^+^-dependent AA absorption across groups in the present vs. the previous study, predominantly attributable to B^0^AT1.

In the intestine, B^0^AT1 associates with ACE2 for functional membrane integration [24]. Besides its role in the renin–angiotensin–aldosterone system, ACE2 has also important functions in inflammatory processes [25] and is the receptor for human severe acute respiratory syndrome corona virus II (SARS-CoV-2) [68]. Infection with SARS-CoV-2 leads to ACE2 internalization [69,70] with retraction of B^0^AT1 from the apical membrane and AA malabsorption [27]. In the present study, we could not identify a decrease in *ACE2* expression, at least at the mRNA level.

Contrary to B^0^AT1 expression, the expression of ATB^0,+^ was reduced in the present study, especially in the DL-Met-supplemented group. Moreover, the level of ATB^0,+^ gene expression was relatively low in the small intestine (average C_t_ values ~ 33), coherent with the finding that ATB^0,+^ mRNA is predominantly expressed in the large intestine [17]. The low expression of ATB^0,+^ was previously used to question the role of ATB^0,+^ in the small intestinal absorption of cationic AAs like Lys [14]. It, thus, remains to be determined whether ATB^0,+^ is the molecular identity of the small intestinal transporter for Na^+^-dependent absorption of cationic AAs or whether another transporter exists with an appropriate substrate spectrum.

As the pig is an excellent model for human gastrointestinal physiology, the results of the present study also carry expectable transferability to humans. However, the study is limited in its use of only one gastrointestinal section. Further studies should attempt to substantiate and refine the proposed model of jejunal AA transport and possibly extend it to other small intestinal sections. Furthermore, it was proposed that the gastrointestinal physiology can differ between males and females, for example, regarding the composition of luminal fluids or gastrointestinal motility [71]. Therefore, studies in female pigs are warranted to elucidate the possible sex differences in AA transport.

## 5. Conclusions

The present study investigated the effects of different Met supplements on the intestinal transport of L-Met and nine other AAs in the porcine small intestine. It showed that a significant part of all tested AAs is transported in a Na^+^-dependent manner in all three Met supplements used. Results were compatible with a model where B^0^AT1 carried a major part of neutral (especially large neutral) AAs with further contributions of an acutely inducible ASCT2 and possibly ATB^0,+^. The proposed transporters were possibly not functional in an earlier study where a diet severely deficient in Met + Cys was supplemented with L-Met or DL-HMTBA. In that previous study, only pigs receiving a DL-Met supplement showed Na^+^-dependent AA transport in their jejunum, which was likely attributable to ASCT2. Animals of the previous trial additionally had a higher inflammatory status in the intestine, which may or may not have been related to the low supply of Met + Cys from true protein and/or the concurrently high levels of Met supplements. Taking these facts together, it appears advisable to prefer DL-Met over other Met supplements if diets require high levels of Met supplementation or if intestinal inflammation occurs in order to preserve Na^+^-dependent AA absorption in the jejunum. With this conclusion, the present study further broadens the knowledge base on AA transport in the context of practical nutrition of pigs with expectable transferability to humans.

## Figures and Tables

**Table 1:** Feed ingredients and calculated nutrient composition of the basal diet (%, as fed basis) ^1^.

Ingredients	% as Fed Basis
Corn	65.41
Soybean meal, 48% CP	21.96
Rapeseed meal	5.88
Corn starch	2.00
Dicalcium phosphate 19	1.81
Soybean oil	0.71
Salt (NaCl)	0.68
L-Lysine sulfate, 54.6% L-Lys ^3^	0.58
Premix Blank Swine ^2^	0.50
Limestone (CaCO_3_)	0.28
L-Threonine ^3^	0.14
L-Tryptophan ^3^	0.04
L-Valine ^3^	0.01
Dry matter	88.87
Calculated nutrient composition	
Crude protein	18.00
Crude fiber	2.72
Ether extract	3.73
Ash	5.90
ME (MJ/kg)	13.78
NE (MJ/kg)	10.40
SID ^4^ Lys	1.07
SID Met	0.25
SID Met + Cys	0.50
SID Thr	0.70
SID Trp	0.21
SID Val	0.73
SID Ile	0.63
SID Leu	1.31
SID Phe	0.75
SID His	0.41
Calcium	0.70
Phosphorus	0.66

^1^ The basal diet was either fed as is (control) or was supplemented with one of three different Met sources: 0.15% DL-Methionine ^3^ (99%; MetAmino), 0.15% L-Methionine ^3^ (99%) or 0.23% DL-HMTBA (free acid, 88%; ALIMET, Novus International, Chesterfield, MO, USA). The amount of corn starch was adjusted to each diet (as fed basis): 1.85% in the DL-Met and L-Met groups and 1.77% in the DL-HMTBA group. ^2^ Premix supplies (per kg of feed): retinyl acetate, 10.000 IU; cholecalciferol, 2.000 IU; DL-α-tocopherol, 40 mg; menadione; 1.5 mg; thiamin, 1.0 mg; riboflavin, 4.0 mg; pyridoxin-HCl, 1.5 mg; cyanocobalamin, 20 µg; niacin, 30 mg; D-pantothenic acid, 15 mg; choline chloride, 150 mg; folic acid, 0.4 mg; biotin, 0.05 mg; Fe (as FeSO_4_·H_2_O), 100 mg; Cu (as CuSO_4_·5H_2_O), 20 mg; Mn (as MnO), 30 mg; Zn (as ZnSO_4_·H_2_O), 70 mg; I (as KI), 0.7 mg; and Se (as Na_2_SeO_3_), 0.25 mg. ^3^ Evonik Operations GmbH, Hanau, Germany. ^4^ SID = standardized ileal digestibility.

**Table 2:** Protein and amino acid contents of basal and experimental diets based on analyzed values (%, as fed basis).

Nutrient Composition	Control	DL-Met	L-Met	DL-HMTBA
Crude protein	17.7	18.8	18.3	18.3
Crude fiber	2.5	2.6	2.6	2.6
Ether extract	4.0	3.7	3.6	3.7
Ash	5.2	5.2	5.1	5.2
Neutral detergent fiber	9.6	9.9	9.6	10.1
Acid detergent fiber	3.5	3.8	3.8	3.8
Starch	45.0	44.4	44.6	45.1
Sugar	3.6	3.8	3.7	3.6
Dry Matter	88.4	88.1	88.2	88.0
Met	0.27	0.43	0.42	0.27
Cys	0.30	0.32	0.31	0.31
Met + Cys	0.57	0.75	0.73	0.58
Lys	1.21	1.29	1.20	1.23
Thr	0.81	0.83	0.82	0.80
Arg	1.12	1.18	1.15	1.12
Ile	0.72	0.75	0.74	0.71
Leu	1.45	1.51	1.47	1.46
Val	0.84	0.87	0.85	0.83
His	0.45	0.47	0.46	0.45
Phe	0.80	0.84	0.82	0.82
Gly	0.73	0.77	0.76	0.74
Ser	0.83	0.86	0.85	0.84
Pro	1.11	1.12	1.10	1.07
Ala	0.88	0.91	0.90	0.89
Asp	1.64	1.73	1.69	1.66
Glu	3.06	3.19	3.12	3.08
Ammonia	0.37	0.39	0.38	0.37
Supplemented				
Met	<0.01	0.15	0.15	<0.01
Lys	0.32	0.32	0.30	0.32
Thr	0.15	0.14	0.14	0.15
Val	<0.02	<0.02	<0.02	<0.02
DL-HMTBA				0.22

**Table 3:** Primer and probe sequences used for RT-qPCR analysis of transporter and cytokine expression.

Gene	Primer	Primer Sequence	Probe Sequence	Accession No	Reference
B^0^AT1 (*SLC6A19*)	Forward	CTTCATCTTCACCCTGAACTC	CCCCTGCTCATCATCGCCTTCTTCGAGATGT	XM_003359855.4	[17]
	Reverse	GATGTCGCTGTTGAACCTG			
ATB^0,+^ (*SLC6A14*)	Forward	CTGTGGCTTGGGGTGGTTTA	CCAACTCCCAGGTGGGCCAT	NM_001348402.1	[17]
	Reverse	AACCAAGCAGCAACCCAAAG			
ASCT2 (*SLC1A5*)	Forward	CGATTCGTTCCTGGATCTTG	CTCCAACCTGGTGTCTGCAGCCTT	XM_003127238.4	[17]
	Reverse	TAGGACGTCGCGTATGAG			
IMINO (*SLC6A20*)	Forward	TCGTGTCCCTCATCAACAG	ACCTCCATCTTTGCCAGTGTCGTCACCTT	XM_003358406.4	[17]
	Reverse	AGGAAGCCATCTTCAAGGTC			
*ACE2*	Forward	ATGGTCGAAAAGTGGTCTGC	GTGCACAAAAGTGACGATGG	NM_001123070.1	
	Reverse	TTCTGAGCAGGTAGGGCTGT			
*CASP1*	Forward	CTCTCCACAGGTTCACAATC	None	NM_214162	[42]
	Reverse	GAAGACGCAGGCTTAACTGG			
*NLRP3*	Forward	AGCACATTCCAGTGCATCAAAG	None	NM_001256770.2	[42]
	Reverse	CCTGGTGAAGCGTTTGTTGAG			
*IL1β*	Forward	CCTCCTCCCAGGCCTTCTGT	None	NM_214055.1	[42]
	Reverse	GGGCCAGCCAGCACTAGAGA			
*IL8*	Forward	GGCAGTTTTCCTGCTTTC	None	X61151.1	[42]
	Reverse	CAGTGGGGTCCACTCTC			
*IL18*	Forward	ACGATGAAGACCTGGAATCG	None	AF191088.1	[42]
	Reverse	GCCAGACCTCTAGTGAGGCTA			
*TGFβ*	Forward	TGACCCGCAGAGAGGCTATA	None	NM_214015.2	[42]
	Reverse	CATGAGGAGCAGGAAGGGC			
*TNFα*	Forward	TTCCAGCTGGCCCCTTGAGC	None	NM_214022.1	[43]
	Reverse	GAGGGCATTGGCATACCCAC			
*GAPDH*	Forward	CAAGAAGGTGGTGAAGCAG	TGAGGACCAGGTTGTGTCCTGTGACTTCAA	XM_021091114.1	[17]
	Reverse	GCATCAAAAGTGGAAGAGTGAG			
*YWHAZ*	Forward	AAGAGTCATACAAAGACAGCAC	ATCGGATACCCAAGGAGATGAAGCTGAA	XM_005662949.2	[17]
	Reverse	ATTTTCCCCTCCTTCTCCTG			

**Table 4:** Correlation coefficients between Na^+^-dependent flux rates of the ten investigated amino acids irrespective of diet of *n* = 37–40 pigs.

	Gln	Gly	Leu	Lys	Met	Ser	Thr	Trp	Tyr	Val
50 µM amino acid concentration										
Gln	---	0.28	0.61 **	0.32	0.42 **	0.42 **	0.51 **	0.30	0.37 *	0.59 **
Gly		---	0.30	0.29	0.30	0.25	0.44 **	0.10	0.31	0.23
Leu			---	0.47 **	0.47 **	0.49 **	0.28	0.27	0.44 **	0.48 **
Lys				---	0.42 *	0.56 **	0.52 **	-0.17	0.70 **	0.11
Met					---	0.58 **	0.31	-0.02	0.44 **	0.55 **
Ser						---	0.45 **	0.02	0.79 **	0.47 **
Thr							---	-0.26	0.58 **	0.12
Trp								---	-0.15	0.29
Tyr									---	0.32 *
Val										---
5 mM amino acid concentration										
Gln	---	0.12	0.40 *	---	0.64 **	0.62 **	0.69 **	---	0.48 **	0.46 **
Gly		---	0.09	---	0.40 *	0.11	0.13	---	0.30	0.05
Leu			---	---	0.58 **	0.32 *	0.19	---	0.32	0.52 **
Lys ^1^	---	---	---	---	---	---	---	---	---	---
Met				---	---	0.36 *	0.58 **	---	0.51 **	0.51 **
Ser				---		---	0.75 **	---	0.14	0.45 **
Thr				---			---	---	0.26	0.42 **
Trp ^1^	---	---	---	---	---	---	---	---	---	---
Tyr				---				---	---	0.37 *
Val				---				---		---

*/** Stars indicate significance of correlation at *p* ≤ 0.05/0.01, respectively. ^1^ Data removed because Na^+^-dependent transport was not significant for Lys and Trp at 5 mM amino acid concentration. Gln, L-glutamine; Gly, glycine; Leu, L-leucine; Lys, L-lysine; Met, L-methionine; Ser, L-serine; Thr, L-threonine; Trp, L-tryptophan; Tyr, L-tyrosine; and Val, L-valine.

## Data Availability

The datasets used and/or analyzed during the current study are available from the corresponding author on reasonable request due to contractual restrictions.
